# Towards an accurate rolling resistance: Estimating intra-cycle load distribution between front and rear wheels during wheelchair propulsion from inertial sensors

**DOI:** 10.1080/02640414.2024.2353405

**Published:** 2024-05-16

**Authors:** Marit P. van Dijk, Louise I. Heringa, Monique A.M. Berger, Marco J.M. Hoozemans, DirkJan H.E. J. Veeger

**Affiliations:** a Department of BioMechanical Engineering, Delft University of Technology, Delft, The Netherlands; b Assistive Technology for Mobility & Sports, The Hague University of Applied Sciences, The Hague, The Netherlands; c Department of Human Movement Sciences, Vrije Universiteit Amsterdam, Amsterdam, The Netherlands

**Keywords:** Rolling resistance, drag force, inertial measurement unit, mechanical power, wheelchair sports

## Abstract

Accurate assessment of rolling resistance is important for wheelchair propulsion analyses. However, the commonly used drag and deceleration tests are reported to underestimate rolling resistance up to 6% due to the (neglected) influence of trunk motion. The first aim of this study was to investigate the accuracy of using trunk and wheelchair kinematics to predict the intra-cyclical load distribution, more particularly front wheel loading, during hand-rim wheelchair propulsion. Secondly, the study compared the accuracy of rolling resistance determined from the predicted load distribution with the accuracy of drag test-based rolling resistance. Twenty-five able-bodied participants performed hand-rim wheelchair propulsion on a large motor-driven treadmill. During the treadmill sessions, front wheel load was assessed with load pins to determine the load distribution between the front and rear wheels. Accordingly, a machine learning model was trained to predict front wheel load from kinematic data. Based on two inertial sensors (attached to the trunk and wheelchair) and the machine learning model, front wheel load was predicted with a mean absolute error (MAE) of 3.8% (or 1.8 kg). Rolling resistance determined from the predicted load distribution (MAE: 0.9%, mean error (ME): 0.1%) was more accurate than drag test-based rolling resistance (MAE: 2.5%, ME: −1.3%).

## Introduction

Rolling resistance is an important resistive force in hand-rim wheelchair propulsion and can vary significantly between wheelchairs, tyres and surface types (de Klerk et al., 2020; Ott & Pearlman, 2021; Rietveld et al., 2021; van der Woude et al., 2003). Accurately assessing rolling resistance is crucial to estimate power output (de Klerk et al., 2020; van Dijk et al., 2024) or to optimize wheelchair settings (Ott & Pearlman, 2021). For this assessment, a drag or deceleration test is commonly used. During a drag test, the force required to pull a wheelchair across a surface is measured. Likewise, during a deceleration test, the wheelchair is accelerated to an initial velocity (at which air resistance can still be assumed negligible) and subsequently passively decelerated. When the deceleration is known and the wheelchair user does not move, the rolling resistance can be determined from this deceleration and the total mass. However, recent studies (van Dijk MP et al., 2024; van Dijk et al., 2024) report a difference between rolling resistance during propulsion and that obtained during these commonly used tests.

**Figure 1.**
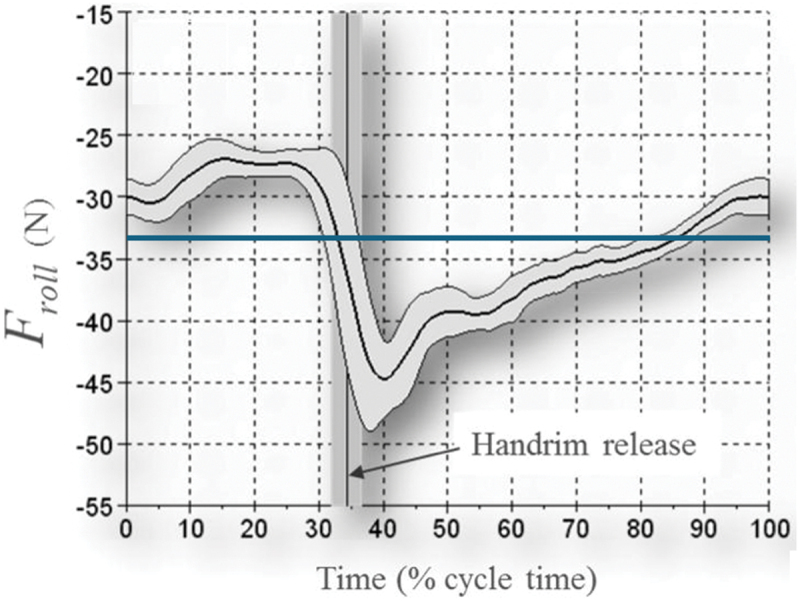
Image of “actual” rolling resistance (black line) and rolling resistance based on a static drag test (blue line). This image is adapted from the image reported by Sauret et al. (2013). Note that – in this example – the drag test-based rolling resistance is similar to the average “actual” rolling resistance. However, depending on the upper body pose during deceleration or drag test, this value may be higher or lower.

The difference between rolling resistance during propulsion and that obtained during drag or deceleration tests can be explained by considering a four-wheeled wheelchair, which is typically used for court sports or everyday use (neglecting the anti-tip wheels, which only sporadically hit the ground), and a wheelchair user who actively moves the upper body during propulsion. As, in such wheelchairs, the front wheels are smaller than the rear wheels, the front wheels have a higher rolling resistance. Due to this difference, inclining the (relatively heavy) trunk – which will shift the mass forward and accelerate the centre of mass vertically – causes the rolling resistance to vary within a push cycle. In addition to upper body motion, a backward “tipping over” moment – that mainly occurs during wheelchair acceleration (van Dijk, van der Slikke, et al., 2021) – may also influence the load distribution between the rear and front wheels. Since intra-cyclical changes in load distribution are neglected by drag and deceleration tests, rolling resistance estimates based on these tests have been found to deviate up to 6% from the actual rolling resistance (Sauret et al., 2013; van Dijk MP et al., 2024) (see Figure 1). *Note that the extent of upper body movement during wheelchair propulsion varies greatly among wheelchair users due to differences in trunk impairment* (Altmann et al., 2015) *or environmental demands. Therefore, the aforementioned deviation is only present and relevant for users and athletes who actively move their trunk during wheelchair propulsion*.

As previous studies reported that a 30% difference in tyre pressure in wheelchair tennis resulted in a 3.3% difference in rolling resistance (Rietveld et al., 2021), and a power difference (determined from rolling resistance times velocity) up to 7% between different wheel configurations (Mason et al., 2015), a deviation of 6% is too much to accurately calculate power output or optimal wheelchair settings. Therefore, more accurate rolling resistance estimates are needed.

To estimate rolling resistance accurately, a continuous determination of load distribution is required. This might be done by measuring the vertical force on the (front and/or rear) wheelchair wheels. However, implementing force sensors is complex, expensive and not sufficiently robust for wheelchair (sports) practice. An alternative is to measure kinematics and derive the instantaneous load distribution from that. Whereas marker-based motion capturing has previously been used for this (Chénier et al., 2023; van Dijk et al., 2024), and inertial sensors (inertial measurement units [IMUs]) are preferred as they are less invasive, inexpensive and readily used in wheelchair (sports) practice. Upper body motions can be approximated by IMU-based trunk motion (ignoring arm movements), and wheelchair kinematics can be obtained from a wheel-mounted IMU (van der Slikke et al., 2015; van Dijk et al., 2022; van Dijk, Kok, et al., 2021). However, a model that predicts load distribution from (IMU-based) trunk and wheelchair kinematics is yet to be developed.

The first aim of this study was to investigate the accuracy of using trunk and wheelchair kinematics to predict the instantaneous load distribution, more particularly front wheel loading, during straight-line hand-rim wheelchair propulsion in a four-wheeled wheelchair. With this prediction, a more accurate (and instantaneous) estimate of rolling resistance may be obtained. Therefore, the second aim of this study was to compare the accuracy of rolling resistance determined from the predicted load distribution with the accuracy of drag test-based rolling resistance. In addition, the robustness of this method was investigated for variations in wheelchair and subject characteristics and trunk use. If the method appears to be accurate and robust, it can be applied in each wheelchair (sport) situation to obtain accurate rolling resistance estimates and, eventually, e.g., power output.

## Materials and methods

### Data collection protocol

**Figure 2.**
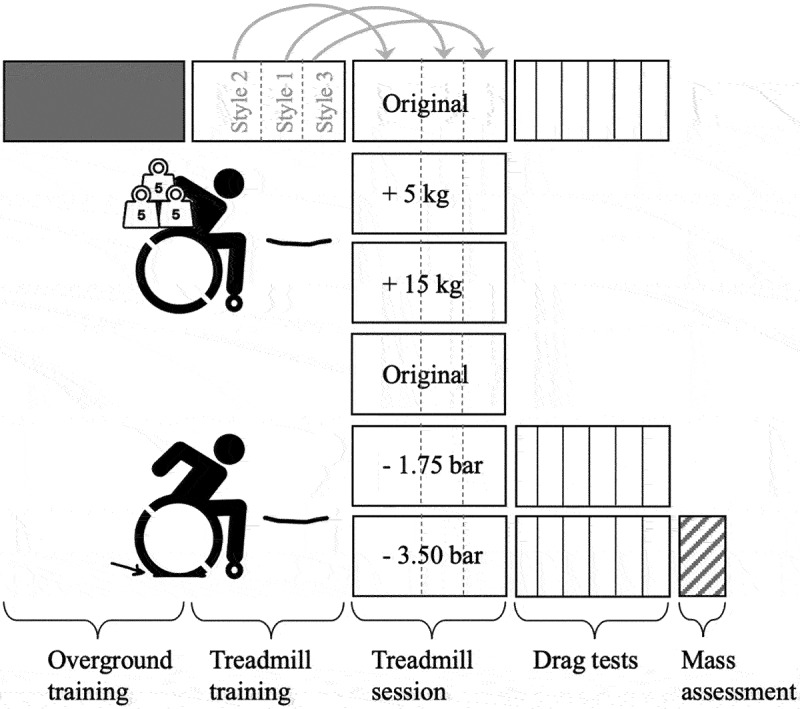
Schematic overview of measurements during different sessions. The “original” treadmill session refers to the condition with no added mass (0 kg) and fully inflated rear wheel tyres (5.25 bar). Mass (i.e., total mass of participant and wheelchair) was assessed on a 1.0 × 1.0-m force plate.

Experimental data from a previous study on wheelchair propulsion were used in this study (van Dijk MP et al., 2024). Twenty-five able-bodied participants (19 females, mean (SD) age: 30 (11) years, mean body mass: 68 (11) kg, body height: 170 (7) cm) with no wheelchair experience were included in the study. Participants propelled the hand-rims of a wheelchair on a large (3.0 × 5.0 m) motor-driven treadmill, while their kinematics were measured with three IMUs (attached to the participant’s sternum, the wheelchair’s frame and right wheel axle), and the front-wheel load was measured using custom-made load pins (in both front wheel axes). Before the treadmill sessions, participants received a 10-min overground wheelchair training to get familiar with the wheelchair and a 10-min training on the treadmill (see Figure 2). In addition, drag tests were performed on the treadmill to obtain rolling resistance coefficients of the (small) front and (large) rear wheels.

To simulate different wheelchair characteristics and pushing styles, the treadmill session was repeated 6 times with different tyre pressures (1.75 bar, 3.5 bar, 5.25 bar) (de Groot et al., 2013) or added mass (0 kg, 5 kg, 15 kg) (van der Slikke et al., 2018, see Figure 2) and with three pushing styles (no trunk motion at 1.2 m · s^−1^ [style 1], unrestricted trunk motion at 1.2 m · s^−1^ [style 2], unrestricted trunk motion at 1.7 m · s^−1^ [style 3] (Marie et al., 2014; Veeger et al., 1989)). By following a metronome (25 beats · min^−1^ in pushing style 2 and 40 beats · min^−1^ in pushing styles 1 and 3), participants were encouraged to make effective pushes, which – in styles 2 and 3 – were accompanied by forward–backward trunk motion. Each treadmill session consisted of 30-s familiarization to the new situation, after which participants propelled 60 s in each pushing style. In this way, a dataset was composed of 18 (three pushing styles and 6 treadmill sessions) 60-s time trials per participant. The order of the treadmill sessions differed per participant.

Drag tests were performed at 1.7 m · s^−1^, while the participants were instructed to sit as still as possible for a period of 30 s in 6 conditions to evoke varying load distributions. The drag test conditions consisted of sitting with an upright trunk and sitting bent forward while no mass was added, while 10 kg was added at the footrests and while 10 kg was added on the upper legs. The drag test-based rolling resistance forces, measured by an S-beam load cell, were obtained by averaging the final 10 s of each drag test condition. Subsequently, the rear (r) and front (f) wheel rolling resistance coefficients, $c_{r}$ and $c_{f}$, were numerically determined by solving Equation 2 based on the average drag force (which equals $F_{\mathrm{IR}}$ in static situations) and average load pin force ($F_{N,f}$) of the series of drag tests, which is similar to previous studies (Bascou et al., 2013; Sauret et al., 2012). Accordingly, $c_{f}$ and $c_{r}$ were used to estimate the gold standard rolling resistance during all treadmill sessions. $F_{N,\mathrm{tot}}$ was assumed to be equal to total mass times 9.81 m · s^−2^.

This study was approved by the ethical committee of Delft University of Technology (Nr. 1530), and written informed consent was obtained from all participants prior to data collection.

(1) $$F_{N,\mathrm{tot}}=F_{N,f}+F_{N,r}$$

(2) $$F_{\mathrm{IR}}=c_{f}*F_{N,f}+c_{r}*F_{N,r}$$

### Instrumentation

All treadmill measurements took place on a large (3.0 × 5.0 m) motor-driven treadmill (Bonte, Zwolle, the Netherlands) at the Vrije Universiteit Amsterdam. A large treadmill was used to make participants feel safe to move forwards, backwards and sideways on the belt. An S-beam load cell (Revere Transducers, Lisse, the Netherlands) was used to measure the horizontal (drag) forces during the drag tests. An RGK Chrome all-courts wheelchair (13.5 kg, camber angle of 13°) was used for the measurements. Load pins (Batarow Sensorik, Germany) were integrated in the front wheel axes of the wheelchair to measure the vertical load on the front wheels. Three IMUs (NGIMU, X-io Technologies, Colorado Springs, CO, USA) were used to collect 3D inertial sensor data with a sample frequency of 100 Hz. In addition, the NGIMU analog input channels of the frame-mounted sensor were connected to the load pins to act as power source and data logger. The load cell was calibrated with known masses at the start of each measurement day. The load pins were calibrated before each measurement session by positioning the front wheels on a custom-made 1.0 × 1.0-m strain gauge force plate (while the rear wheels were positioned on a dummy plate at the same height) (Kingma et al., 1995).

### Predicting load distribution between front and rear wheels

#### Preprocessing

After data collection, the force plate calibration data were used to convert voltage of the two individual load pins to vertical force. Subsequently, the summed vertical force on the front wheels was normalized to percentage of the total vertical force on all 4 wheels (i.e., total mass times gravitational force). As vertical accelerations of the upper body were previously shown to have no effect on rolling resistance (van Dijk MP et al., 2024), vertical accelerations were assumed to be zero.

In addition, predictor features that represented different aspects of trunk and wheelchair motion were generated based on IMU data (see Table 1). Subsequently, all data were second-order low-pass filtered with a cut-off frequency of 3 Hz. This cut-off frequency was chosen based on the assumption that load is influenced mainly by trunk motion, which has a maximal frequency of around 2 Hz. The last 60 s in each pushing style were analysed.

**Table 1.** Overview of the abbreviations and calculation method of all predictor features and outcome feature that were used in the predictive model.

	Predictor feature	Determined by
$v_{\mathrm{wc}}$	Wheelchair velocity in m/s^−1^	(Gyroscope signal of IMU around wheel axis ⋅ rear wheel diameter ⋅ π)/360 (van der Slikke et al., 2015)^a^
$a_{\mathrm{wc}}$	Wheelchair acceleration in m/s^−2^	Derivative of $v_{\mathrm{wc}}$
$\varphi_{\mathrm{tr}}$	Trunk inclination angle in rad	Based on extended Madgwick filter (van Dijk, Kok, et al., 2021), with β-value being 0.0015 (if |wheelchair acceleration| <0.1 m · s^−2^ for at least five consecutive samples) or 0.9635 (otherwise)
${\dot{\varphi }}_{\mathrm{tr}}$	Angular velocity of trunk (around sagittal axis) in rad/s^−1^	Gyroscope signal (around sagittal axis) of trunk-mounted IMU
φ tr	Angular acceleration of trunk (around sagittal axis) in rad/s^−2^	Derivative of ${\dot{\varphi }}_{\mathrm{tr}}$
$a_{\mathrm{tr},⊥}$	Trunk acceleration perpendicular to the frontal plane of the trunk in m/s^−2^	Acceleration signal (directed perpendicular to frontal plane) of trunk-mounted IMU
$a_{\mathrm{tr},∥}$	Trunk caudal-cranial acceleration in m/s^−2^	Acceleration signal (in caudal-cradial direction) of trunk-mouned IMU
$\left|a_{\mathrm{tr}}\right|$	Magnitude of trunk acceleration vector in m/s^−2^	Euclidean norm of the three-dimensional acceleration signal of the trunk-mounted IMU subtracted by 9.81
	**Outcome feature**	**Determined by**
${\hat{F}}_{N,f}$	Normalized relative front wheel-load in %	Force data from the front wheels’ load pins, calculated as $F_{N,f}$/$F_{N,\mathrm{tot}}$ × 100%

^a^In case of curves and turns, the linear velocity obtained from the wheel should be corrected for turning using the algorithm described by van der Slikke et al. (2015).

To generate the final dataset, all treadmill session data were sequenced into one large dataset consisting of the “outcome” feature (relative front wheel load, or ${\hat{F}}_{N,f}$), “predictor” features ($v_{\mathrm{wc}}$, $a_{\mathrm{wc}},\varphi_{\mathrm{tr}}$, ${\dot{\varphi }}_{\mathrm{tr}},\;\varphi \;\mathrm{tr}$, $a_{\mathrm{tr},⊥}$, $a_{\mathrm{tr},∥}$, $\left|a_{\mathrm{tr}}\right|$) and “descriptive” variables (participant number, pushing style and session type) (see Table 1). The outcome and predictor features were standardized to a z-score. Since the best predictive model could contain linear or non-linear relations, both linear methods and (non-linear) machine learning methods were examined for predicting the relative front wheel load from the predictor features.

#### Training, validation and test set

From the full dataset, three random participants and two test configurations (+5 kg and −1.75 bar, see Figure 2) were removed to act as “test” set for model evaluation in a later stage. The reduced dataset based on 22 participants and 4 configurations was split randomly – while each treadmill session was kept in the same set – a training set (80% or 66 treadmill sessions) and a validation set (20% or 17 treadmill sessions). The training set was used to determine the best set of predictive features to predict the relative front wheel load (${\hat{F}}_{N,f}$) and to “train” all models. The validation set was used to select the best model and the best model hyperparameters. Finally, the test set was used once the final model was obtained to determine how well the model performs on unseen data (i.e., data that were not previously used to train the model or to make decisions). This was done by comparing the predicted relative front wheel load (and resulting rolling resistance) with the measured relative front wheel load (and resulting rolling resistance). The test set was assumed to be representative for any new dataset.

#### Feature, model and hyperparameter selection

As the inclusion of outcome-unrelated features may result in overfitting, a feature selection was performed to select the best feature combination. In this study, an exhaustive feature selection method based on a random forest regressor (RFR) and 7-fold cross-validation (leaving one-seventh of the subsets out during each iteration) was used. An RFR was used as this model is fast and robust to overfitting. According to the results of the exhausting feature selection, the best trade-off between maximal accuracy (more features) and chance of overfitting (less features) was found with three features: linear wheelchair velocity, linear wheelchair acceleration and linear acceleration perpendicular to the trunk (i.e., $v_{\mathrm{wc}}$, $a_{\mathrm{wc}}$ and $a_{\mathrm{tr},⊥}$, see Table 1).

With the aforementioned three-feature combination, five different model types were trained on the training set: a simple linear regression (LR), an RFR, a multilayer perceptron (MLP), a long short-term memory (LSTM) and a gate recurrent unit (GRU) models. The LR model was used to investigate whether a linear relation may be used to solve the relation between trunk and wheelchair motion and front wheel-load. The RFR has been known as a robust and fast algorithm and may therefore be suitable to predict front wheel-load. In addition, three (deep) neural networks have been tested. LSTM and GRU models both take previous time samples into account, which might yield good results as well. MLP, LSTM and GRU have all been proven useful to predict ground reaction forces from IMU data in walking and running (Alcantara et al., 2022; Davidson et al., 2019; Leporace et al., 2015, 2018; Pogson et al., 2020; Sharma et al., 2021). After training, the most predictive model was selected by applying the five models to the validation set data and determining the mean error (ME; i.e., the mean difference between the observed and predicted relative front wheel load), mean absolute error (MAE) and root-mean-squared error (RMSE). The model with the overall best performance was assumed to be the best model type. To maximize performance of the final model, hyperparameters were tuned based on the validation set. Lastly, the best model type with selected hyperparameters was trained again based on all data from the training set. The predictions from the final model were evaluated hereafter. Based on the final model, the relative front wheel load was predicted for all test set data.

### Evaluating predicted load distribution

#### 1– How well does the final model predict relative front wheel load?

To determine the accuracy of the model predictions, the relative front wheel load was predicted for all test set data and compared with the measured relative front wheel load. Differences between the two values were expressed in ME, MAE and RMSE. To assess whether the model was prone to overfitting, a second version of the final model was obtained based on the training dataset and a Gaussian noise layer that was added to the original model architecture. A large difference between the results of both models indicates that the model’s output is influenced by white noise on the input data, and thus overfits the data.

##### 2 – Does adding a trunk-mounted IMU and a prediction model result in a more accurate rolling resistance estimate than drag test-based rolling resistance estimates?

To convert proportions (%) to absolute normal forces, the proportions were multiplied by $F_{N,\mathrm{tot}}$, and subsequently, rolling resistance was calculated according to Equations 1 and 2. To evaluate whether the model improves the rolling resistance estimates, the predicted rolling resistance was compared to the “gold standard” load pin-based rolling resistance. Secondly, the predicted rolling resistance was compared to the drag test-based rolling resistance. Therefore, the drag test-based rolling resistance was determined based on the rolling resistance coefficients that were determined previously and the load distribution corresponding to the drag tests in upright position.

##### 3 – Is the model robust for different wheelchair characteristics and pushing styles?

To evaluate model robustness, the final prediction model was used to predict load distribution in situations that were not used to train the model, with *only* the participants who were not included to train the model (i.e., unseen conditions and unseen subjects, so 6 treadmill sessions). Subsequently, these load distributions were used to determine rolling resistance force. ME, MAE and RMSE were reported for different propulsion styles and for different wheelchair configurations (averaged over three “unseen” participants).

## Results

**Figure 3.**
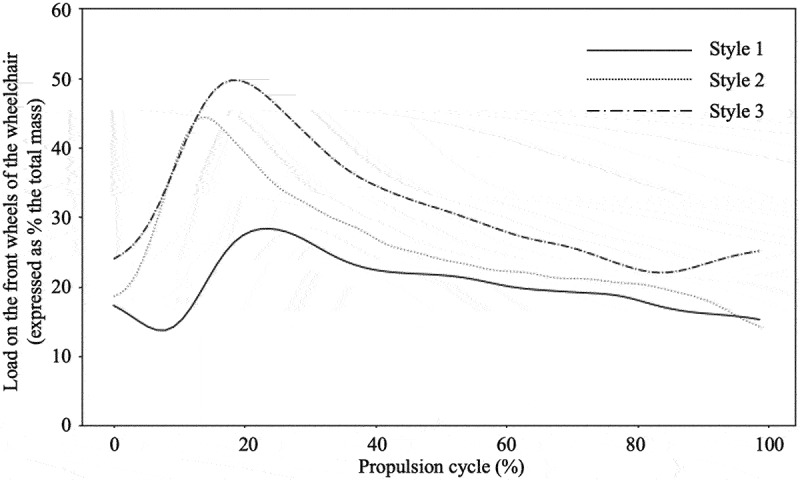
Typical example of the relative load on the front wheels of the wheelchair (expressed as percentage of the total mass of the wheelchair and user) of a representative subject in the original wheelchair condition during one propulsion cycle (0% representing the start of the push) for propulsion style 1 (no trunk motion at 1.2 m · s^−1^), style 2 (unrestricted trunk motion at 1.2 m · s^−1^) and style 3 (unrestricted trunk motion at 1.7 m · s^−1^).

In the present study, 143 treadmill sessions were included, of which 83 were used to train and select the final machine learning model (i.e., training and validation set) and 60 were used to evaluate the results (i.e., the test set). Seven sessions were left out due to incompleteness (caused by empty batteries or connection errors). As expected, the largest variation (and largest value) in front wheel load was found for the third pushing style that was characterized by the largest trunk motion (see Table 2 and Figure 3). The average front wheel load differed considerably between participants (see “Range” in Table 2). The total mass (participant + wheelchair) in the test set was on average 76.9 ± 9.1 kg. Overall, the test set seems a decent reflection of the training dataset.

**Table 2.** Mean (variation) and range of relative front wheel loads (expressed as percentage (%) of the total load) for all participants per pushing style (characterized by the amount of trunk motion, i.e., TM) in the training and validation set and test set. The range consists of the average front wheel load of the participant with the lowest load and the average front wheel load of the participant with the highest load. The variation is determined by the average of the standard deviations per person. The values are based on 60 s of propulsion per pushing style per person.

	Style 1: No trunk motion	Style 2: Moderate trunk motion	Style 3: Full trunk motion
Mean (training and validation set) (%)	20.0 (4.9)	24.9 (6.9)	27.1 (9.2)
Range (training and validation set) (%)	12.8–26.3	14.0–32.1	17.5–35.4
Mean (test set) (%)	20.7 (4.4)	24.0 (6.4)	24.9 (7.6)
Range (test set) (%)	15.2–26.2	16.3–31.5	17.0–31.2

### 1 – How well does the final model predict relative front wheel load?

Exhaustive feature selection resulted in wheelchair velocity, wheelchair acceleration and trunk acceleration (perpendicular to the frontal plane of the trunk) being the most predictive features for relative front wheel loading. With these features, five different model types were trained. Overall, LSTM turned out to yield the most accurate predictions (see Table 3). The hyperparameter combination that resulted in the lowest MAE value consisted of one hidden layer, 50 neurons, a learning rate of 0.01, a batch size of 128, a dropout rate of 0.1 and 20 time steps. This final model showed an MAE of 3.8% ± 1.8% relative front wheel load corresponding to about (68 kg × 3.8) 2.6 ± 1.2 kg on an average compared to the actual measured load on the front wheels and an RMSE of 4.4% ± 1.6% (see Table 3).

**Table 3.** The results in terms of mean error (ME), mean absolute error (MAE) and root-mean-squared error (RMSE) of the five model types that were trained to predict the front wheel load as a percentage of the total mass of wheelchair and user (i.e., RFWL). Models were trained on the training set (with default hyperparameters) and evaluated on the validation set for which the results are shown.

	Evaluated on validation set					Evaluated on test set	
	LR	RFR	MLP	LSTM	GRU	LSTM (final)	LSTM (final) + noise
ME (RFWL)	1.9	1.8	1.6	1.0	1.2	0.5	0.5
MAE (RFWL)	4.4	3.8	3.4	2.7	2.9	3.8	3.8
RMSE (RFWL)	5.9	5.1	4.6	3.5	3.6	4.4	4.4
Comp. time E^−03^ (s)	0.0	0.1	0.4	1.8	1.8	-	-

The final model and the second version of the final model in which a Gaussian noise layer was added resulted in comparable accuracies (see right side of Table 3), indicating that the model does not overfit the data.

### 2 – Does adding a trunk-mounted IMU and a prediction model result in a more accurate rolling resistance estimate than drag test-based rolling resistance estimates?

**Figure 4.**
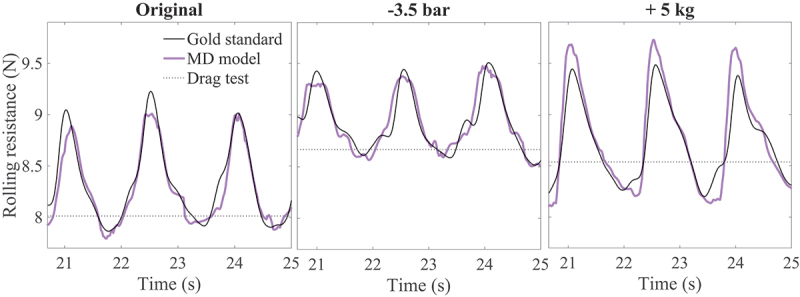
Gold standard rolling resistance (black line), load distribution model-based rolling resistance (purple line) and drag test-based rolling resistance (dotted line) for the “original” condition, condition with −3.5 bar tyre pressure and condition with +5 kg added mass.

As the third pushing style (unrestricted trunk motion at 1.7 m · s^−1^) showed larger variations in normal force and has previously been shown to deviate more from drag test-based rolling resistance estimates, data are reported for the third pushing style in Table 4. The rolling resistance estimates based on the final (LSTM) model had a similar shape as those based on the gold standard load pin force (Figure 4). Whereas drag test-based rolling resistance tends to be underestimated (ME of −1.3), with the application of the prediction model, these underestimations were absent (ME of 0.1). A similar trend was seen for MAE and RMSE (see Table 4).

**Table 4.** Comparisons between the accuracies of the load distribution model-based rolling resistance (F_roll,LD model_) and the drag test-based rolling resistance (F_roll,drag_) based on the “original” condition-data from the test set (pushing style: full trunk motion). Accuracies were determined by comparison with the “gold standard” load pin-based rolling resistance (F_roll,gold standard_) and expressed in mean error (ME), mean absolute error (MAE) and root-mean-squared error (RMSE). The actual gold standard rolling resistance values are also reported. The measures are given for each subject in the test set.

	F_roll,gold standard_ (N)	F_roll,LD model_ − F_roll,gold standard_ (%)			F_roll,drag_ − F_roll,gold standard_ (%)		
		ME	MAE	RMSE	ME	MAE	RMSE
Subject 1	8.6 (1.0)	0.5	0.7	0.9	−1.1	1.4	1.8
Subject 2	9.9 (1.0)	−0.4	1.0	1.3	−0.4	3.3	4.0
Subject 3	9.4 (0.9)	0.3	1.0	1.4	−2.3	2.8	3.9
Mean	9.3	0.1	0.9**	1.2*	−1.3	2.5	3.2

* *p* < 0.05, ** *p* < 0.01.

### 3 – Is the model robust for different wheelchair characteristics and pushing styles?

Looking at different wheelchair characteristics and pushing styles, again, the deviations with the gold standard are considerably smaller for the prediction model compared to the drag test-based rolling resistance (see Figure 4 and Table 5). The errors are similar for the different conditions. Therefore, the model seems to be robust for different unseen characteristics.

**Table 5.** Comparisons between the accuracies of the load distribution model-based rolling resistance (F_roll,LD model_) and the drag test-based rolling resistance (F_roll,drag_) based on the “−3.5 bar” and “+5 kg” condition-data from the test set (for each pushing style). Accuracies were determined by comparison with the “gold standard” load pin-based rolling resistance (F_roll,gold standard_) and expressed in mean error (ME), mean absolute error (MAE) and root-mean-squared error (RMSE). The actual gold standard rolling resistance values are also reported.

Pushing style	F_roll,gold standard_ (N)	F_roll,LD model_ − F_roll,gold standard_ (%)			F_roll,drag_ − F_roll,gold standard_ (%)		
		ME	MAE	RMSE	ME	MAE	RMSE
**−3.5 bar condition**							
Style 1	8.5 (0.7)	−0.5	0.8	1.0	0.4	1.5	1.9
Style 2	8.7 (0.8)	−0.4	1.0	1.3	−1.1	2.3	2.8
Style 3	8.7 (0.8)	−0.1	1.0	1.3	−1.2	2.7	3.4
**+5 kg condition**							
Style 1	8.4 (0.5)	0.2	1.1	1.3	1.6	2.7	3.3
Style 2	8.5 (0.5)	−0.1	1.5	2.0	−0.2	3.2	3.8
Style 3	8.6 (0.6)	0.2	1.4	1.8	−0.9	3.7	4.5

## Discussion

The aim of this study was to investigate the accuracy of using trunk and wheelchair kinematics to predict the instantaneous load distribution, more particularly front wheel loading, during hand-rim wheelchair propulsion, such that – eventually – rolling resistance estimates could be improved. Based on two inertial sensors (one at the trunk and one at the wheelchair wheel), and a machine learning model (which is publicly accessible – see Appendix A), the front wheel load could be predicted up to 2.6% (or 1.8 kg) accuracy (MAE). When this front wheel load is subsequently used to estimate rolling resistance, rolling resistance estimates have an accuracy of about 0.9% (MAE) and ME of 0.1%, which was significantly lower than the rolling resistance estimates without the model. Moreover, the robustness of the model was tested for different wheelchair characteristics and pushing styles. As the accuracy did not differ between different wheelchair characteristics and pushing styles (i.e., MAE ranging from 0.8 to 1.5 and ME ranging from −0.5 to 0.2), we assumed the model to be robust for different circumstances.

The average front wheel loads in the present study (24.9% ± 6.9%) were lower than the average front wheel loads of about 40% reported by Sauret et al. (2013) and Brubaker (1986). This may have to do with the different types of wheelchairs used. In wheelchairs designed for everyday use, stability of the wheelchair (not falling over) is often regarded as more important than the rolling resistance of the wheelchair (by limiting the weight on the front wheels), such that the seat (and thus the centre of mass) is put more forward deliberately. Hence, sports wheelchairs typically exhibit lower rolling resistances in comparison to everyday-use wheelchairs. Besides these differences, the front wheel load development over a push cycle in the present study is similar to those reported by Sauret et al. (2013). Overall, our results are well in line with previously reported values and patterns of front wheel load.

Based on the results of the present study, the front wheel load can continuously be determined during wheelchair propulsion. In this way, the effects of upper body motion and wheelchair accelerations on rolling resistance can be incorporated such that accurate rolling resistance estimates can be obtained. As the presented model makes the rolling resistance values sensitive to different circumstances (i.e., large vs. small upper body motions and high vs. low accelerations), the estimates are more accurate and more individualized than rolling resistance values determined based on drag or deceleration tests.

An important implication of this higher accuracy and more individualized rolling resistance is that comparisons between and within wheelchair athletes are much fairer. Traditional drag test-based estimates lead to unfair comparisons, especially when trunk motion varies between athletes. For instance, during a sprint, the initial pushes involve higher rolling resistance due to acceleration and larger trunk inclination, contrasting with periods of constant velocity with less trunk inclination. Ignoring these variations (as is done during deceleration or drag tests) results in inaccurate power comparisons within a wheelchair sports team. The present load distribution model offers more accurate estimates of rolling resistance and power, ensuring fairer comparisons between and within wheelchair users and athletes compared to traditional drag test-based methods.

For wheelchair (sports) practice, the presented method is ready to be applied. When trunk and wheelchair kinematics are obtained (e.g., using inertial sensors), and a set of deceleration tests are performed to obtain the rolling resistance coefficients, accurate estimates of rolling resistance can be determined. Subsequently, accurate rolling resistance estimates can be used to monitor mechanical power or to optimize wheelchair set-up and/or tyre pressure. However, it should be noted that the improved rolling resistance estimates with the prediction model come with extra information that is required about trunk motion and a machine learning model that should be executed. As the error based on drag or deceleration tests only – without incorporating changes in load distribution – is on average 3% (see right side of Table 4) and differs from 1% to 6% (as reported in our previous study (van Dijk MP et al., 2024)), this may be accurate enough for some purposes such as for recreational wheelchair sports or everyday wheelchair use. Therefore, depending on the required accuracy, one may choose to base the rolling resistance on drag or deceleration tests only – instead of applying the load distribution model – and accept some inaccuracies.

### Limitations

For this study, some limitations should be noted. First, wheelchairs with different front-to-rear wheel distances or different seat positions were not tested in the present study. However, as front-to-rear wheel distances are assumed to differ <20 cm in general, and a similar front wheel load pattern was observed in the study of Sauret et al. (2013) with a “general use” wheelchair, we assume the model is suitable for other wheelchair dimensions. Second, the participants who were tested in the present study had no disabilities. The model should ideally be tested with wheelchair users with different movement strategies or different physique (e.g., missing body parts) to assess whether the model is robust for movement strategies (e.g., non-symmetrical trunk motion) used by this group. Lastly, participants in this study had no wheelchair experience, which may affect their propulsion technique. However, the estimation of load distribution is expected to be independent of propulsion technique as it reflects trunk motion and the resulting centre of mass displacement. Therefore, the experience level of participants will not influence the translatability of our model to experienced wheelchair users.

## Conclusion

In hand-rim wheelchair propulsion, the estimation of the continuous intra-cyclic load distribution between front and rear wheels could be determined with an accuracy of 3.8% MAE (or 1.8 kg) based on two inertial sensors and a machine learning model. Rolling resistance determined from the predicted load distribution (MAE: 0.9%, ME: 0.1%) was more accurate than the rolling resistance based on drag tests only (MAE: 2.5%, ME: −1.3%). Since the model is based on a relatively large number of participants, a considerable variation in front wheel load between participants, and different wheelchair characteristics and pushing styles, the model is considered valid to estimate rolling resistance in a wide range of wheelchair (sports) situations and for a wide range of wheelchair users.

## Data Availability

The raw data supporting the conclusions of this article are publicly available at 10.4121/bc9a8588-5e50-4dff-aa77-5114ff7626f7.v2. The machine learning model, including instructions on how to use it, is available at 10.4121/c533f919-1a44-48d5–8543-5c7f8be29bb0.

## Appendix A: Instruction on how to apply the LD model on kinematic data

Step-by-step explanation on how to collect and pre-process IMU data, use the LD model to predict load distribution and export the full dataset to a CSV file is presented. The dataset used for the current study is publicly available at https://data.4tu.nl/datasets/bc9a8588-5e50-4dff-aa77-5114ff7626f7.

1. Download model from: https://data.4tu.nl/authors/60e44636-eb8f-43f9-a9f4-436bfde4872c

2. Collect kinematic data

- of coast-down tests with different (known) load distributions

- of the situation/actions of interest (if IMUs are used to collect the data, attach one IMU to the wheelchair wheel axle, one IMU to the centre of the wheelchair frame and one IMU around the chest against the sternum)

3. Determine from the data:

The rolling resistance coefficients per pair of wheels

- The acceleration vector perpendicular to the trunk

- The wheelchair velocity

- The wheelchair acceleration

- Time

4. Save the above-mentioned variables in the following order:

i.e., df[“samples”,“time”,“v_wc”, “a_wc”, “trunk_acc_3”]

5. Run the code below in Python

6. Done

“””

Created on Fri Nov 17 14:12:28 2023

Predict Relative Front Wheel Load (RFWL) during wheelchair propulsion

@author: L.H.A. Heringa & M.P. van Dijk

“””

import pandas as pd

import numpy as np

import matplotlib.pyplot as plt

from keras.models import load_model

# Run functions

def standardize(signal):

size_temp = np.asarray(signal.shape)

size = size_temp[0].item()

standarized_signal =np.zeros((size,3))

i = 0

while i < 3:

z_score = (signal[:,i]-mean[i])/std[i]

standarized_signal[:,i] = z_score

i = i + 1

return standarized_signal

def load(x_data, num_steps):

#x_data = data.iloc[:,[2,3,4]].to_numpy()

X = list() #reshape the 2d data into 3d sliding window shape

for i in range(x_data.shape[0]):

end_ix = i + num_steps # compute a new (sliding window) index

if end_ix ≥ x_data.shape[0]: # if index is larger than the size of the dataset, stop

break

seq_X = x_data[i:end_ix] # Get a sequence of data for x

x. append(seq_X)

x_array = np.array(X)

return x_array

# Load model

model = ”/./final_lstm.h5”

# Load IMU data

df_full = pd.read_csv(r’/./IMU_Data.csv’)

df = df_full[[“samples”,“time”,“v_wc”, “a_wc”, “trunk_acc_3”]].to_numpy()

mean = np.mean(df, axis = 0)

std = np.std(df, axis = 0)df_new = standardize(df[:,2:5])

# Run and plot LSTM model

num_steps = 20

X = load(df_new,num_steps)

lstm = load_model(model)

y = lstm.predict(X)

plt.plot(y)

#Add force output to initial dataset

RFWL_Output = pd.DataFrame(y)

df_full_new = df_full[num_steps:df_full.shape[0]]

df_full_new[“RFWL_Output”] = y

# Export dataset to csv

df_full_new.to_csv(r’/./IMU_Data_plus_predictedFrontWheelLoad.csv’)
